# Physical activity in the rural population of Pelotas, Brazil: prevalence and associated factors

**DOI:** 10.11606/S1518-8787.2018052000265

**Published:** 2018-09-13

**Authors:** Rafaela Costa Martins, Inácio Crochemore Mohnsam da Silva, Pedro Curi Hallal

**Affiliations:** I Universidade Federal de Pelotas. Faculdade de Medicina. Departamento de Medicina Social. Pelotas, RS, Brasil

**Keywords:** Adult, Exercise, Activities of Daily Living, Cross-Sectional Studies, Rural Population, Adulto, Exercício, Atividades Cotidianas, Estudos Transversais, População Rural

## Abstract

**OBJECTIVE:**

To evaluate the level of physical activity in general and by domains of practice in the rural area of Pelotas, State of Rio Grande do Sul, Brazil, as well as their associated factors.

**METHODS:**

This is a population-based, cross-sectional study with adults living in the rural area of Pelotas. The questionnaire used to measure the prevalence of physical activity was the Global Physical Activity Questionnaire. Individuals who reported at least 150 minutes of weekly physical activity were considered as active. The demographic, economic, labor, and crime safety aspects were evaluated as independent variables. Poisson regression was used for the crude and adjusted analyses.

**RESULTS:**

Final sample consisted of 1,447 individuals. Overall prevalence of physical activity was 83.7% (95%CI 81.3–86.2). Regarding the different domains, 74.9% (95%CI 71.3–78.6) of the participants reached the recommendations of physical activity specifically with work, 25.2% (95%CI 22.4–28.0) with transport, and 15.1% (95%CI 12.2–18.1) with leisure. Men were more active than women in all domains. Individuals with rural work were more active in work and transport. Crime variables were not associated with outcomes.

**CONCLUSIONS:**

The prevalence of general physical activity was high, and was mostly practiced at work. On the other hand, leisure activities were not very prevalent and the associated factors varied in direction and magnitude according to the domains of physical activity evaluated.

## RESUMO

**OBJETIVO:**

Avaliar o nível de atividade física geral e por domínios de prática na zona rural de Pelotas, RS, bem como seus fatores associados.

**MÉTODOS:**

Estudo transversal de base populacional realizado com adultos moradores da zona rural de Pelotas. O questionário utilizado para mensurar a prevalência de atividade física foi o *Global Physical Activity Questionnaire*. Foram considerados como ativos os indivíduos que relataram pelo menos 150 minutos de prática de atividade física semanal. Aspectos demográficos e econômicos, laborais e de segurança contra crimes foram avaliados como variáveis independentes. A regressão de Poisson foi utilizada para as análises bruta e ajustada.

**RESULTADOS:**

A amostra final foi composta por 1.447 indivíduos. A prevalência geral de atividade física foi de 83,7% (IC95% 81,3–86,2). Quanto aos diferentes domínios, 74,9% (IC95% 71,3–78,6) dos participantes atingiram as recomendações de atividade física especificamente no trabalho, 25,2% (IC95% 22,4–28,0) no deslocamento e 15,1% (IC95% 12,2–18,1) no lazer. Os homens foram mais ativos que as mulheres em todos os domínios. Os indivíduos com situação ocupacional rural foram mais ativos no trabalho e no deslocamento. As variáveis de crime não foram associadas aos desfechos.

**CONCLUSÕES:**

A prevalência de atividade física geral foi alta, e majoritariamente praticada no trabalho. Por outro lado, as atividades de lazer foram pouco prevalentes e os fatores associados variaram em direção e magnitude de acordo com os domínios de atividade física avaliados.

## INTRODUCTION

Physical inactivity is a risk factor for several chronic diseases. More than 5.3 million deaths per year worldwide are attributed to physical inactivity^1^. Even so, almost a quarter of the adult population and more than half of older adults^2^ do not reach the recommendations of 150 minutes of weekly physical activity (PA)^3^. Because of the harmful effects of physical inactivity on health and the low levels of physical activity of the world population, physical inactivity has received the status of pandemic^4^.

Most studies are carried out in urban areas, and there is little evidence on the levels of PA and their associated factors in rural populations, which represents 46% of the world population^5^ and 16% of the Brazilian population^6^. Given the sociodemographic, economic, environmental, labor, and behavioral differences between urban and rural areas, it is expected that both the prevalence of physical inactivity and the factors influencing the practice of PA are different between these population groups. The economy of Pelotas’ rural area is based on cattle breeding and the production of peach, tobacco, and rice, except for one district, which is focused on fishing. The distance between houses and social cohesion are considerably higher in the rural than in the urban area. Most of the work activities require physical labor. Also, leisure activities are different from the urban area, which indicates possible differences in behavior (for example in PA). The physical environment (natural or built), for example, is associated with the level of PA of populations, as well as exposure to crime, transportation systems, and urbanization^7^. In addition to sociodemographic factors, all these aspects are distributed differently in the urban and rural areas, especially in middle- and low-income countries. Therefore, the accumulation of evidence based on urban areas cannot be extrapolated directly to the entire population.

The Brazilian National Health Survey (PNS)^8^ has shown that the prevalence of general physical inactivity in the rural area (48.3%) was higher than that found for the urban population (45.6%). A population-based study conducted in the rural area of State of Minas Gerais, Brazil, using the same definition of physical inactivity as our article, has identified prevalence equal to 13.5%^9^. In addition to the lack of studies in this population, mainly in Brazil, where there is only one population-based study, there is still a gap on the prevalence of physical inactivity in individuals living in rural areas, as well as their associated factors. These data are essential to improve health diagnosis and establish public interventions and policies in a population group that is historically left behind by the scientific community. Thus, the objective of this study was to evaluate the level of PA in general and by domains in the rural area of Pelotas, State of Rio Grande do Sul, Brazil, as well as their associated factors.

## METHODS

We carried out a population-based study as a research consortium, which brings together various health interests. With a cross-sectional design, this study was carried out between January and July 2016 in the rural area of Pelotas, State of Rio Grande do Sul, Brazil, in its eight districts with approximately 22,000 inhabitants. The main characteristics of the region are the predominance of small rural properties with rice plantation and fourteen basic health units distributed among the districts. We considered eligible all individuals aged 18 years or more who usually lived in the household or who were absent for a period not exceeding 12 months.

To calculate the sample size estimated by OpenEpi, we considered a prevalence of 86.5%, error of three percentage points, significance of 5%, and effect of delineation of 2.0, which resulted in 1,217 participants. Sampling was performed by clusters and established in two stages. First, of the 50 census tracts in the rural area of Pelotas, 24 were randomly selected with probability proportional to the number of households with permanent residents of each district. According to the Brazilian Institute of Geography and Statistics (IBGE), Brazil has approximately two adults per residence; therefore we defined that 30 houses from each census tract should be selected to reach the sample size. In this selection of households, we used Google Earth to identify the centers, that is, agglomerates with the largest number of households [at least five houses close to each other (up to 1 km)]. Each center had a core – a place with more streets connected – and the houses were selected randomly from one of the directions of the streets. If we did not identify 30 households in the first center, we started the search for the second center with the largest number of households, and so on.

To measure the outcome, trained interviewers applied the Global Physical Activity Questionnaire (GPAQ) to the participants, measuring PA in three domains (work, transport, and leisure). This questionnaire was submitted to a repeatability and concurrent validity study in a city that was emancipated 22 years ago from the rural area of Pelotas (n = 50, estimated based on an intraclass correlation coefficient of 0.79), which still has rural characteristics. The intraclass correlation coefficient between two applications of the questionnaire in the one-week interval was 0.78 (95%CI 0.63–0.87). We found moderate correlation (r = 0.5) between the weekly minutes of moderate and vigorous PA from the questionnaire when compared to the objective measurement using accelerometry (article in press). The outcome was dichotomized, and we considered active the individual who reached, in a usual week, at least 150 minutes of moderate PA, 75 minutes of vigorous PA, or the equivalent combination of both intensities. In addition, we considered the level of PA in each domain as the outcome and the same operational definition was used.

The independent variables evaluated were sex (male; female), categorical age in complete years (18–24 years; 25–39 years; 40–59 years; 59 years or more), categorical education level (0–4 years; 5–8 years; 9 years or more), occupation in three categories (no work, rural work, non-rural work), body mass index (BMI), calculated by the division between weight and height squared (low weight or eutrophic; overweight; obesity), marital status (whether or not living with a partner), and socioeconomic level, based on the index of goods measured by principal component analysis. Rural work was defined as specific work in the rural area, such as farming, field work, care of animals, use of tractors, plantations, among others.

In addition to the sociodemographic characteristics, we evaluated the perception of crime safety and victimization as potential environmental factors associated with the practice of PA according to a scale based on the Neighborhood Environment Walkability Scale (NEWS) and the City Stress Inventory (CSI). The variable of perceived crime safety considered as poor the participants who answered “once” or “more than once” for some of the following questions: 1) “Since <month> last year, how many times has a sale or purchase or drugs happened near your home?”; 2) “Since <month> last year, how many times has a burglary taken place in your neighborhood, that is, something was taken without violence or threat?”; 3) “Since <month> last year, how many times has physical assault occurred between people in your neighborhood?”; 4) “Since <month> last year, how many times has a robbery taken place in your neighborhood, that is, something was taken by violence or threat?”; 5) “Since <month> last year, how many times has murder happened in your neighborhood?”. In relation to victimization, the questions used were: 1) “Since <month> last year, how many times have you been the victim of burglary, that is, any of your belongings were taken without violence or threat?”; 2) “Since <month> last year, how many times have you been a victim of robbery, that is, any of your belongings were taken by violence or threat?”; 3) “Since <month> last year, how many times have you been the victim of physical assault from someone other than your family?” If the participant answered “once” or “more than once” to one of the questions, they were considered a victim.

Data analysis was carried out in the statistical package Stata, version 12. Sampling was by clusters and the *svy* command was used for weight, for the under- or overrepresentation of households in the district. The proportion of each variable was presented, in addition to their respective 95%CI. Comparison of proportions between groups was done by the chi-square test of heterogeneity. A linear trend was tested by Poisson regression when it had an apparent linear association. When the linear trend test did not present a significant value, the p-value presented in the tables was for heterogeneity. Crude and adjusted analyses were performed by Poisson regression^10^. We used a four-level hierarchical conceptual model for the adjusted analysis. In the first level, we inserted the variables of sex and age; in the second level, we inserted the variables of education, occupation, and marital status; in the third level, we inserted the variable of income; and, in the last level, we inserted the variables of perception of crime safety, victimization, and BMI. In the regression, the variables were inserted into the model using the backwards selection, level by level, excluding those variables with p < 0.20. After adjusting for each level, the variables of the previous levels remained in the model regardless of the p value^11^ ^,^ ^12^. No collinearity was found between the variables of the model after analysis of the inflation value of the variance. The quality of fit of the model was ensure with Pearson testing and model quality deviation.

The project was approved by the Ethics Committee of the Faculdade de Medicina of the Universidade Federal de Pelotas (Process 1,363,979). All individuals signed the informed consent. More details on the methodology can be found in the article of Gonçalves et al.^13^

## RESULTS

Of the 1,697 eligible individuals, we had 178 losses and refusals, resulting in a total sample of 1,519 persons. In addition to these losses, 72 individuals did not answer all questions of the PA questionnaire, amounting to 1,447 persons in the final sample. Men accounted for 64% of the losses and refusals. The response rate of this study was 85.3%. Most of the sample consisted of women (51.7%), those aged between 40 and 59 years (39.2%), those with lower education level (38.7%), those not working (40.6%), those overweight (35.3%), those who lived with a partner (70.8%), those with a good perception of crime safety (74.5%), and those who were not victims of burglary, robbery, or physical assault from someone other than family in the last 12 months (94.5%) (Table 1). The overall prevalence of active individuals was 83.5% (95%CI 81.0–85.9). Evaluating the different domains, prevalence was 74.9% (IC95% 71.3–78.6) for work, 25.2% (95%CI 22.4–28.0) for transport, and 15.1% (95%CI 12.2–18.1) for leisure (Figure). All categories of the variables of sex, occupation, and index of goods were more prevalent in the work domain (Figure).

**Table 1:** Description of the sample and distribution of the variables of exposure according to active individuals. Pelotas, State of Rio Grande do Sul, Brazil, 2016.

Variable	n (%)	Level of physical activity (%)	95%CI	p^b^
Sex				0.025
Male	734 (48.3)	600 (85.8)	83.1–88.4	
Female	785 (51.7)	612 (81.8)	78.5–85.1	
Age (years)				< 0.001
18–24	174 (11.4)	152 (90.7)	86.4–94.9	
25–39	341 (22.6)	285 (86.2)	81.5–90.8	
40–59	593 (39.2)	500 (88.3)	84.9–91.7	
60 or over	411 (26.8)	275 (71.7)	67.9–75.5	
Years of study				< 0.001
0–4	582 (38.7)	422 (78.1)	74.9–81.4	
5–8	558 (36.9)	468 (86.9)	82.7–91.0	
9 or more	369 (24.4)	316 (87.8)	83.6–92.0	
Occupation				< 0.001
Not working	613 (40.5)	417 (72.4)	68.2–76.6	
Rural	509 (33.3)	469 (95.9)	94.5–97.3	
Non-rural	397 (26.2)	326 (85.2)	82.1–88.4	
Body mass index^a^				< 0.001^c^
Low weight/Eutrophic	499 (35.1)	426 (88.3)	85.3–91.4	
Overweight	509 (35.3)	417 (85.1)	81.0–89.1	
Obesity	425 (29.6)	330 (80.1)	76.1–84.2	
Living with a partner				0.032
No	443 (29.2)	329 (79.2)	73.9–84.5	
Yes	1,076 (70.8)	883 (85.5)	82.8–88.3	
Index of goods (quintiles)				0.002
1st (poorest)	303 (20.4)	210 (74.4)	69.2–79.6	
2nd	302 (19.9)	237 (83.0)	78.4–87.6	
3rd	302 (20.0)	254 (88.3)	84.4–92.2	
4th	301 (19.8)	252 (86.8)	81.2–92.3	
5th (richest)	301 (19.9)	250 (85.7)	80.6–90.7	
Perception of safety				0.386
Good	1,129 (74.5)	895 (83.2)	80.2–86.2	
Poor	390 (25.5)	317 (85.2)	81.5–89.0	
Crime victimization				0.963
No	1,437 (94.5)	1,145 (83.7)	81.4–86.0	
Yes	82 (5.5)	67 (84.0)	71.6–96.3	

Total	1,519	1,447 (83.7)		

^a^ Variable with most missing data (n = 1,433).

^b^ Chi-square test of heterogeneity.

^c^ Test for linear trend.

**Figure:**
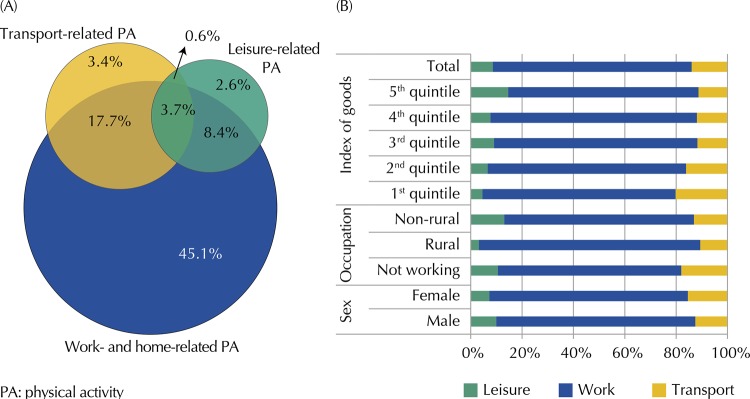
(A) Venn diagram with the proportion of individuals according to level of physical activity in each domain and (B) proportion of physical activity by domains in relation to total physical activity according to sex, occupation, and index of goods. Pelotas, State of Rio Grande do Sul, Brazil, 2016. (n = 1,447)

Considering all domains of PA, males, individuals aged 18 to 24 years, those with nine or more years of study, workers (mainly those with rural work), those with normal or low BMI, those who lived with a partner, and those belonging to the 3rd income quintile had a higher prevalence of PA (Table 1). Table 2 presents the prevalence of PA in the domains according to the independent variables. Males were more active than females in all domains (p < 0.001), and this difference was more marked in transport and leisure-related PA (approximately 10 percentage points). Younger individuals were more active in the domain of leisure, and those aged 40–59 were more active in the domain of work and transport. In transport-related PA, there was no difference between education levels; in leisure-related PA, those with higher education level were more active; in work-related PA, those with education level between five and eight years were the most active. The most active were those in the highest quintile of income (24.2%) for leisure, those belonging to the fourth income quintile (29.0%) for transport, and those in the fourth income quintile (79.8%) for work. Participants who did not live with a partner were more active in the domain of leisure and those who lived with a partner were more active in the domain of work. Individuals with rural work were more active in the domain of work and transport; however, those with non-rural work were the most active in the domain of leisure (22.6%). Individuals not working were the least active in all domains.

**Table 2:** Distribution of the variables of exposure according to active individuals in the domains of leisure, transport, and work. Pelotas, State of Rio Grande do Sul, Brazil, 2016.

Variable	Leisure-related PA (n = 1,498)			Transport-related PA (n = 1,486)			Work-related PA (n = 1,461)		
		
	n (%)	95%CI	p	n (%)	95%CI	p	n (%)	95%CI	p
Sex			< 0.001			0.003			0.027
Male	147 (20.5)	15.8–25.2		209 (29.0)	25.9–32.1		546 (77.4)	73.2–81.6	
Female	79 (10.2)	8.1–12.3		168 (21.6)	17.8–25.5		550 (72.7)	68.5–76.8	
Age (years)			< 0.001			0.002			< 0.001
18–24	57 (33.3)	23.8–42.7		40 (23.6)	17.0–30.2		131 (78.3)	17.0–30.2	
25–39	58 (17.3)	12.5–22.0		81 (24.0)	19.7–28.2		263 (78.6)	71.9–85.4	
40–59	71 (11.9)	8.6–15.3		179 (30.2)	25.8–34.6		468 (81.8)	78.0–85.6	
60 or over	40 (10.2)	6.8–13.5		77 (19.5)	15.6–23.3		234 (60.1)	55.5–64.7	
Years of study			< 0.001*			0.916			0.008
0–4	48 (8.5)	5.3–11.6		141 (25.0)	21.7–28.3		388 (70.3)	66.4–74.2	
5–8	87 (15.7)	11.8–19.7		144 (25.7)	21.3–30.1		429 (79.2)	74.3–84.2	
9 or more	88 (24.1)	17.0–31.3		91 (25.2)	20.5–29.8		273 (75.8)	69.2–82.4	
Occupation			0.002			< 0.001			< 0.001
Not working	72 (12.3)	9.3–15.2		103 (17.6)	14.6–20.5		352 (60.4)	56.4–64.4	
Rural	65 (12.8)	8.6–16.9		186 (36.6)	32.6–40.6		458 (92.4)	90.7–94.2	
Non-rural	89 (22.6)	16.8–28.3		88 (22.2)	16.9–27.4		286 (74.6)	69.7–79.5	
BMI			0.003*			0.113			< 0.001*
Low weight/Eutrophic	93 (18.6)	14.7–22.5		139 (28.1)	24.4–31.7		390 (80.0)	75.3–85.2	
Overweight	81 (16.0)	11.8–20.3		123 (24.3)	20.4–28.1		380 (76.7)	71.8–81.5	
Obesity	49 (11.7)	8.4–15.1		104 (24.3)	20.2–28.4		289 (69.7)	65.1–74.4	
Living with a partner			0.011			0.793			0.002
No	84 (19.5)	14.8–24.3		107 (24.8)	20.0–29.6		283 (67.4)	60.5–74.4	
Yes	142 (13.4)	10.2–16.5		270 (25.4)	22.7–28.0		813 (78.0)	74.7–81.2	
Index of goods (quintiles)			< 0.001			0.162			< 0.001
1st (poorest)	27 (9.4)	6.2–12.7		71 (24.0)	18.8–29.2		185 (64.8)	59.8–69.8	
2nd	32 (10.8)	7.3–14.3		84 (28.4)	22.7–34.1		223 (76.6)	72.6–80.7	
3rd	53 (17.9)	11.6–24.2		74 (24.7)	18.6–30.7		230 (79.4)	73.1–85.7	
4th	39 (13.3)	8.0–18.5		87 (29.0)	22.8–35.3		233 (79.8)	74.1–85.5	
5th (richest)	73 (24.2)	18.5–29.9		60 (20.4)	15.2–25.6		217 (73.8)	66.4–81.1	
Perception of safety			0.297			0.318			0.356
Good	162 (14.6)	11.5–17.8		270 (24.3)	21.0–27.6		808 (74.4)	70.1–78.8	
Poor	64 (16.6)	12.6–20.6		107 (27.9)	21.4–34.4		288 (76.4)	73.0–79.8	
Victim of crimes			0.123			0.783			0.187
No	207 (14.7)	11.9–17.5		357 (25.3)	22.4–28.1		1,041 (75.4)	72.0–78.8	
Yes	19 (22.7)	9.6–35.8		20 (23.8)	13.2–34.5		55 (67.4)	52.5–82.2	

PA: physical activity; BMI: body mass index

* Test for trend.

After adjustment, the variable of sex remained significant only in the domains of leisure and transport (men were more active than women) and age was the same as in the crude analysis, in which younger individuals were more likely to reach the recommendations of general and leisure-related PA. For leisure, the older the individuals, the less likely they are to be active. When we analyzed all domains of PA, the persons with a higher education level had an 11% higher prevalence of being active when compared to less educated individuals (PR = 1.11; 95%CI 1.05–1.18). The variables of crime were not significant in the crude or adjusted analysis. Marital status and BMI did not remain statistically significant after adjustment in any domain or in the general context (Tables 3 and 4).

**Table 3:** Crude analysis performed by Poisson regression of the prevalences of physical activity in general and by domains in relation to the independent variables. Pelotas, State of Rio Grande do Sul, Brazil, 2016.

Variable	General PA		Leisure-related PA		Transport-related PA		Work-related PA	
			
	PR (95%CI)	p	PR (95%CI)	p	PR (95%CI)	p	PR (95%CI)	p
Sex		0.029		< 0.001		0.003		0.026
Male	Ref.		Ref.		Ref.		Ref.	
Female	0.95 (0.91–1.00)		0.50 (0.41–0.61)		0.75 (0.62–0.90)		0.94 (0.89–0.99)	
Age (years)		< 0.001		< 0.001*		0.001		< 0.001
18–24	Ref.		Ref.		Ref.		Ref.	
25–39	0.95 (0.90–1.00)		0.52 (0.38–0.72)		1.02 (0.73-1.41)		1.00 (0.91–1.10)	
40–59	0.97 (0.92–1.03)		0.36 (0.25–0.51)		1.28 (0.93–1.76)		1.05 (0.95–1.15)	
60 or over	0.79 (0.74–0.85)		0.31 (0.21–0.45)		0.82 (0.55-1.24)		0.77 (0.67–0.88)	
Years of study		< 0.001*		< 0.001*		0.919		0.005
0–4	Ref.		Ref.		Ref.		Ref.	
5–8	1.11 (1.05–1.18)		1.86 (1.16–2.98)		1.03 (0.89–1.19)		1.13 (1.06–1.20)	
9 or more	1.12 (1.06–1.19)		2.85 (1.82–4.44)		1.01 (0.81–1.25)		1.08 (0.99–1.18)	
Occupation		< 0.001		< 0.001		< 0.001		< 0.001
Not working	Ref.		Ref.		Ref.		Ref.	
Rural	1.33 (1.25–1.40)		1.04 (0.70–1.55)		2.08 (1.70–2.56)		1.53 (1.43–1.64)	
Non-rural	1.18 (1.11–1.25)		1.84 (1.39–2.44)		1.26 (0.95–1.68)		1.23 (1.14–1.33)	
BMI		0.001*		0.001*		0.154		0.001*
Low weight/Eutrophic	Ref.		Ref.		Ref.		Ref.	
Overweight	0.96 (0.92–1.01)		0.86 (0.67–1.12)		0.87 (0.73–1.03)		0.96 (0.89–1.04)	
Obesity	0.91 (0.86–0.96)		0.63 (0.49–0.81)		0.87 (0.74–1.02)		0.87 (0.81–0.94)	
Living with a partner		0.047		0.011		0.793		0.006
No	Ref.		Ref.		Ref.		Ref.	
Yes	1.08 (1.00–1.17)		0.68 (0.52–0.91)		1.02 (0.86–1.22)		1.16 (1.05–1.28)	
Index of goods (quintiles)		0.007		0.001		0.224		0.006
1st (poorest)	Ref.		Ref.		Ref.		Ref.	
2nd	1.12 (1.02–1.22)		1.15 (0.76–1.74)		1.18 (0.88–1.60)		1.18 (1.08–1.30)	
3rd	1.19 (1.08–1.30)		1.90 (1.23–2.92)		1.03 (0.75–1.41)		1.23 (1.10–1.36)	
4th	1.17 (1.07–1.27)		1.41 (0.84–2.35)		1.21 (0.87–1.68)		1.23 (1.12–1.36)	
5th (richest)	1.15 (1.07–1.24)		2.56 (1.72–3.82)		0.85 (0.67–1.08)		1.14 (1.04–1.24)	
Perception of safety		0.378		0.297		0.313		0.363
Good	Ref.		Ref.		Ref.		Ref.	
Poor	1.03 (0.97–1.08)		1.14 (0.89–1.45)		1.15 (0.87–1.52)		1.03 (0.97–1.09)	
Victim of crimes		0.963		0.109		0.785		0.257
No	Ref.		Ref.		Ref.		Ref.	
Yes	1.00 (0.87–1.16)		1.54 (0.90–2.65)		0.94 (0.61–1.47)		0.89 (0.73–1.09)	

PA: physical activity; PR: prevalence ratio; BMI: body mass index. Ref.: reference

* Test for trend.

**Table 4:** Adjusted analysis performed by Poisson regression of the prevalences of physical activity in general and by domains in relation to the independent variables. Pelotas, State of Rio Grande do Sul, Brazil, 2015.

Variable	General PA		Leisure-related PA		Transport-related PA		Work-related PA	
			
	PR (95%CI)	p	PR (95%CI)	p	PR (95%CI)	p	PR (95%CI)	p
Sex		0.064		< 0.001		0.004		0.051
Female	Ref.		Ref.		Ref.		Ref.	
Male	1.04 (1.00–1.09)		1.96 (1.60–2.40)		1.33 (1.11–1.61)		1.06 (1.00–1.12)	
Age (years)		< 0.001		< 0.001*		0.002		< 0.001
18–24	Ref.		Ref.		Ref.		Ref.	
25–39	0.95 (0.90–1.01)		0.54 (0.40–0.72)		1.03 (0.74–1.42)		1.01 (0.92–1.11)	
40–59	0.98 (0.93–1.03)		0.37 (0.26–0.52)		1.29 (0.94–1.77)		1.05 (0.95–1.16)	
60 or over	0.79 (0.74–0.85)		0.32 (0.22–0.47)		0.84 (0.56–1.25)		0.77 (0.67–0.89)	
Years of study		0.001*		0.004*		0.224		0.040
0–4	Ref.		Ref.		Ref.		Ref.	
5–8	1.08 (1.02–1.15)		1.51 (0.94–2.44)		1.04 (0.89–1.22)		1.08 (1.01–1.15)	
9 or more	1.12 (1.05–1.19)		1.95 (1.24–3.05)		1.21 (0.97–1.50)		1.08 (1.00–1.16)	
Occupation		< 0.001		0.079		< 0.001		< 0.001
Not working	Ref.		Ref.		Ref.		Ref.	
Rural	1.29 (1.21–1.38)		0.87 (0.55–1.38)		1.85 (1.43–2.38)		1.44 (1.35–1.54)	
Mon-rural	1.11 (1.03–1.20)		1.31 (0.95–1.79)		1.13 (0.80–1.59)		1.14 (1.05–1.23)	
BMI		0.226		0.344		0.599		0.088
Low weight/Eutrophic	Ref.		Ref.		Ref.		Ref.	
Overweight	1.00 (0.95–1.05)		0.95 (0.71–1.27)		0.92 (0.77–1.09)		1.00 (0.93–1.09)	
Obesity	0.95 (0.90–1.01)		0.81 (0.61–1.09)		0.95 (0.80–1.12)		0.93 (0.86–1.00)	
Living with a partner		0.205		0.448		0.302		0.082
No	Ref.		Ref.		Ref.		Ref.	
Yes	1.05 (0.97–1.14)		0.90 (0.68–1.19)		0.90 (0.73–1.11)		1.09 (0.99–1.21)	
Index of goods (quintiles)		0.140		0.009*		0.153		0.241
1st (poorest)	Ref.		Ref.		Ref.		Ref.	
2nd	1.07 (0.97–1.17)		1.04 (0.69–1.56)		1.09 (0.84–1.42)		1.10 (1.00–1.21)	
3rd	1.12 (1.03–1.21)		1.67 (1.10–2.54)		0.93 (0.69–1.24)		1.11 (1.02–1.22)	
4th	1.08 (1.00–1.17)		1.15 (0.70–1.87)		1.09 (0.82–1.45)		1.10 (1.00–1.21)	
5th (richest)	1.07 (0.99–1.15)		1.96 (1.22–3.15)		0.79 (0.64–0.98)		1.03 (0.96–1.12)	
Perception of safety		0.972		0.824		0.347		0.577
Good	Ref.		Ref.		Ref.		Ref.	
Poor	1.00 (0.95–1.06)		0.97 (0.72–1.30)		1.12 (0.87–1.45)		1.02 (0.96–1.08)	
Victim of crimes		0.517		0.144		0.624		0.362
No	Ref.		Ref.		Ref.		Ref.	
Yes	1.03 (0.94–1.13)		1.44 (0.87–2.38)		0.88 (0.53–1.48)		0.93 (0.80–1.09)	

PA: physical activity; PR: prevalence ratio; BMI: body mass index. Ref.: reference

* Test for linear trend.

## DISCUSSION

The prevalence of physically active individuals found in rural areas was high (83.7%), that is, more than four fifths of the population performed at least 150 minutes of moderate to vigorous PA per week. However, there is a large difference between domain prevalence. Age, years of study, and occupation were the variables associated with general PA. Work-related physical activities are the main responsible for this high percentage of general PA. This result highlights only one of the several differences between rural and urban populations, as work-related PA in urban areas is an increasingly uncommon habit^14^ ^,^ ^15^. The variables that remained associated with PA after adjustment were sex, age, years of study, and occupation. Leisure is a social practice potentially responsible for improving the quality of life because of its physiological health benefits and reduced mortality^16^ ^,^ ^17^. However, in the leisure domain, only 15.1% of the individuals reached the recommended levels. In this study, only the variables of sex, age, years of study, and index of goods were associated with leisure-related PA. For transport-related PA, only the variables of sex, age, and occupation were associated.

Data related to the level of PA in the Brazilian population differ greatly by region, city, or area (rural and urban). Data from the 2013 PNS showed a prevalence of 54% of active individuals, considering both urban and rural environments^18^. In the urban area of Pelotas, the prevalence of PA in the four domains has been decreasing (58.9% in 2002, 48.0% in 2007, and 45.6% in 2012)^19^. In two rural communities of the State of Minas Gerais, Brazil, the prevalence was 86.5%^9^. Although the population of Pelotas lives in a common location, the frequency of PA of the rural inhabitants was much more similar to the frequency of the rural inhabitants of Minas Gerais, which indicates that urban-rural differences are more marked than regional differences in Brazil. Two studies, one in Sri Lanka and the other in Malaysia^19^ ^,^ ^20^, confirm this hypothesis. The rural population, in general, presents higher values of PA than the urban population. This usually happens because work is extremely manual and active. Considering that persons should practice PA in order to improve their quality of life, for pleasure and leisure – in addition to energy expenditure –, the promotion and encouragement of leisure practices, the creation of suitable places for this end, and the availability of professionals who encourage this practice is extremely important in rural areas.

A study carried out in India using the GPAQ has shown prevalence of PA in the urban and rural areas of 35% and 50%, respectively^22^. Another study carried out in Bangladesh has shown a higher prevalence of transport-related PA in the urban area and work-related PA in the rural area; the prevalence of leisure-related PA was extremely low both in urban and rural areas (< 3%)^23^. A great potential of the GPAQ is the greater comparability between studies. In 2017, the questionnaire was used in a Brazilian survey, for example^24^. The prevalence of PA mentioned in this paragraph exemplifies the wide use of the GPAQ in various contexts and emphasizes that the differences found are potentially specific to local realities and less susceptible to problems related to measurement.

Although the association between PA and sociodemographic factors is well established in the literature, the evidence is mostly from urban studies. Exclusive evidence from rural populations is still needed because of the large social, cultural, and economic differences between these contexts. In our study, men were more active than women in leisure and transport. In rural areas of the United States (USA), women are more active than men, which is an important difference in relation to our findings^25^. In rural Bangladesh, a low-middle income country, males are more active than females in relation to general PA (mean of 1,934 minutes and 653 minutes, respectively)^26^. The lack of an association between sex and work-related PA was probably because both women and men work actively using manual labor in rural areas. Therefore, there is no consensus in the literature regarding PA in the rural population, especially in Brazil, whose regions differ in many respects. In addition, we have no population-based study on physical activity in the southern area of the country and only one study in Brazil, which hindered comparability.

We identified that individuals whose occupation involved some rural work did more PA in the domains of work, transport, and general. Only 12.8% of those who had rural work practiced leisure-related PA. This can be explained by the intense labor that rural workers perform, resulting in inactive leisure activities^27^. It is important to note that the health of the rural worker should be given special attention, since regular work happens at a faster pace, often occupying more than half of the day, in addition to this population being exposed to agrochemicals, machinery, and solar radiation^28^ ^,^ ^29^. These characteristics of intense work in the field can cause health problems such as skin cancer, gonalgia, and minor psychiatric disorders^29–31^.

Physical activity is determined by several factors, such as the social environment. The two variables related to crime were selected to be part of the model because violence is increasing in rural areas^32^. However, the perception of crime safety and victimization were not associated with any of the outcomes. The low prevalence of participants who reported feeling unsafe in the rural area of Pelotas resulted in a small sample size for these analyses. Thus, the sample may have been insufficient to identify the association. Individuals in the urban area feel more unsafe than residents of the rural area and they use more resources to protect their homes (extra locks, security bars, alarms). Nevertheless, robberies and burglaries, whether or not they are effective, and physical assaults have increased in both urban and rural areas^32^. In addition, a study carried out in the urban area of Pelotas has found no association between perceived crime safety and PA in adults^33^. If it was not due to a random error, some hypotheses may explain this lack of association, as active transport is often not a matter of choice and the pleasure of practicing leisure-related PA is greater than insecurity, or this practice occurs far from the place of residence^34^.

This study has some limitations. Among them, there is the possibility of reverse causality between the outcome and BMI, because of the cross-sectional design. The crime questionnaire has not been validated in the Brazilian rural population, but it was adapted from the version used in studies in the urban area of Pelotas^33^. In the same way, the so-called asphalt bias may have affected the study, since we selected the houses that were closer to more streets, and fewer houses were likely selected in areas farthest from each center of the tract. Thus, we may have underestimated the level of transport-related PA, since the individual who lives far away probably does not travel on foot. However, the domains of work and leisure do not change with asphalt bias because they are independent of the individual’s place of residence. Another limitation refers to the distribution of losses and refusals, since 64% were male. As men were more active than women, this limitation may have underestimated the percentages of PA.

In addition to the aforementioned factors, the difficult in understanding the questionnaire and the measurement of the time of PA by the interviewees can also be considered a weakness of this study. However, a repeatability and concurrent validity study of the GPAQ performed in the city of Arroio do Padre, a predominantly rural city recently emancipated from Pelotas, has shown that the main limitations of the questionnaire refer to individuals who report a high practice of PA – approximately 400 minutes or more per week^35^. In our study, as the main interest was to identify and describe the participants who reach the recommendations of 150 minutes of PA per week, the application of this questionnaire can be considered adequate.

We can also identify strengths in this study. First, this is a population-based study with a large sample size. In addition, there are few studies on PA in individuals living in rural areas around the world, with only one prevalence study in Brazil^9^. Furthermore, the questionnaire used (GPAQ) is recommended by the World Health Organization (WHO); that is, several studies worldwide use it to determine the prevalence of PA, which increases the comparability of this study.

In conclusion, the prevalence of active individuals in general was high and mostly practiced at work. On the other hand, leisure activities were less practiced (15.1%). These values show that neither the level of physical activity nor its associated factors found in urban areas can be extrapolated to the rural area of the same city. Our results are essential for the proposal of contextualized strategies, which encourage leisure practices and promote quality of life in the rural population.
